# Acceptability of 11 fortified balanced energy‐protein supplements for pregnant women in Nepal

**DOI:** 10.1111/mcn.13336

**Published:** 2022-03-09

**Authors:** Tsering P. Lama, Subarna K. Khatry, Sheila Isanaka, Katie Moore, Leslie Jones, Juliet Bedford, Joanne Katz, Saskia de Pee, Steven C. LeClerq, James M. Tielsch

**Affiliations:** ^1^ Department of International Health Johns Hopkins Bloomberg School of Public Health Baltimore Maryland USA; ^2^ Nepal Nutrition Intervention Project – Sarlahi (NNIPS) Kathmandu Nepal; ^3^ Departments of Nutrition and Global Health and Population Harvard T.H. Chan School of Public Health Boston Massachusetts USA; ^4^ Anthrologica Oxford UK; ^5^ Department of Global Health, Milken Institute School of Public Health George Washington University Washington District of Columbia USA

**Keywords:** acceptability, balanced energy protein supplement, hedonic scale, Nepal, pregnant women, test feeding

## Abstract

Evidence suggests that multiple micronutrient and balanced energy protein (BEP) supplementation during pregnancy can decrease the risk of stillbirth and small‐for‐gestational‐age births and increase birth weight. We conducted a mixed‐methods formative research study to identify the most acceptable among a range of 11 candidates fortified BEP supplements for use in pregnancy and lactation in a rural district in Nepal. Forty pregnant women aged 15–40 years participated in a test meal tasting of 11 different sweet and savoury candidate BEP supplements. Each participant rated the products on organoleptic properties using a 7‐point hedonic scale (1 = Dislike it very much to 7 = Like it very much), ranked her ‘top 3’ most liked supplements, and subsequently discussed each product with peers in focus group discussions (FGDs). Five supplements (sweet lipid‐based nutrient supplement (LNS), savoury LNS, sweet vanilla biscuits, vanilla instant drinks and seasoned pillows) achieved the maximum overall median hedonic score of 7, with sweet LNS and seasoned pillows ranking as the top 2. This was consistent with the assessments in FGDs. Women in the FGDs expressed dislike of the smell and taste of the cocoa drink, savoury masala bar, sweet mango bar and savoury curry biscuit, which was consistent with the hedonic scale scores. This study provides valuable insights into our understanding of women's acceptance of different BEP supplements during pregnancy in rural Nepal and has helped identify the two most accepted BEP supplements to be used in a two‐month home trial to assess utilisation and compliance in this setting.

## Key messages

- Understanding the acceptability of balanced energy protein supplements by pregnant women along with the potential facilitators and barriers to their use is vital to ensure regular consumption and compliance.
- Pregnant women in rural Nepal liked both the sweet and savoury products and suggested having the option to choose from more than one type of product would facilitate its daily consumption throughout pregnancy.
- Concerns regarding the cost of the supplements, strong aversion to smell/taste of certain supplements, and the inability to eat the supplement in private were some of the deterrents for possibly not being able to eat the supplement daily

## Graphical abstract

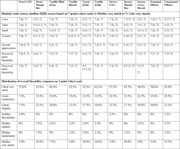

## 1 INTRODUCTION

Maternal undernutrition is highly prevalent in low and middle‐income countries (LMICs) where the increased nutritional requirements during pregnancy are often unmet through traditional diets (S. E. Lee et al., 2013). The first 1000 days of a child's life, from conception until the child's second birthday, is a critical period to lay the foundations for optimal child health, growth and development. The nutritional status of the mother and child during this time can have a profound impact on a child's ability to grow, learn and thrive (Martorell, 2017).

It was estimated that in 2015, one in seven newborns, or 20.5 million babies globally, were born low birth weight (LBW); 48% of these were born in South Asia and 24% in sub‐Saharan Africa (Blencowe et al., 2019). The average annual risk reduction (AARR) has stalled in the past decade to only 1% (Blencowe et al., 2019). The 2025 World Health Assembly target to reduce prevalence of LBW by 30% will be unattainable unless the AARR is tripled to 2.7%, and so there is more need now than ever to better understand the potential for interventions towards reaching these global targets (United Nations, 2015). The high burden of LBW is biologically linked to the high burden of maternal undernutrition. Undernourished women in many LMICs enter pregnancy with low nutritional reserves; an estimated 240 million are underweight (body mass index [BMI] < 18.5; Di Cesare et al., 2016). Low maternal BMI and short stature are associated with increased risks of a variety of adverse reproductive outcomes including preterm birth and small for gestational age (SGA), the two underlying causes of LBW (Kozuki et al., 2015; A. C. C. Lee et al., 2013; Rahman et al., 2015).

One potential nutritional intervention is the current recommendation made by the World Health Organization (WHO) in the 2016 antenatal care guidelines to provide BEP dietary supplementation in undernourished populations (WHO, 2016). This recommendation is based on findings from systematic reviews of BEP supplementation during pregnancy that demonstrate moderate levels of evidence that such dietary supplementation can decrease the risk of stillbirth and SGA births and increase birth weight (Imdad & Bhutta, 2011; Ota et al., 2015; Stevens et al., 2015). The systematic reviews of existing BEP trials, however, showed that the studies used a wide range of BEP supplements, which differed in energy content, nutritional composition and forms (Bill & Melinda Gates Foundation, 2017). In 2016, The Bill & Melinda Gates Foundation (BMGF) convened an expert group to recommend the optimal nutritional composition of a BEP supplement for use in pregnant and lactating women (PLW) in low income and food insecure contexts (Bill & Melinda Gates Foundation, 2017). Before implementing the randomised controlled trial (RCT) designed to test the efficacy of the BEP supplement(s) during pregnancy and lactation on pregnancy and child health outcomes in rural Nepal (ClinicalTrials.gov Identifier: NCT03668977), it is important to first identify which of the BEP supplements is well accepted by the participants in the study area. To optimise effectiveness, the BEP supplement to be used in the RCT must be consumed on a daily basis throughout pregnancy by those in the intervention group. Sensory characteristics of food such as taste, texture, smell and appearance have distinct and influential effects on food acceptability (Piqueras‐Fiszman & Spence, 2015). On the individual level, complaints about the sensory properties of the supplements, particularly during pregnancy, are known to influence adherence as seen in many studies (Clermont et al., 2018; Harding et al., 2017; Klevor et al., 2016; Lutsey et al., 2008; Young et al., 2010). The hedonic scale (quantitively collected) is a widely used method for quantitatively measuring consumers' experienced quality of food (i.e., liking and acceptability; Lawless & Heymann, 2010). Personal factors such as values, beliefs, attitudes and demographics as well as situational factors like meal preparation, consumption situation, context and environment can influence food quality perceptions (Furst et al., 1996). Qualitative methods such as observations, in‐depth interviews and focus group discussions (FGDs) are commonly used to understand the contextual factors influencing quality perceptions. A mixed approach of quantitative and qualitative methods is commonly applied to obtain detailed and nuanced information concerning factors that could affect the acceptability of supplements as evident in several acceptability of nutritional supplements studies among PLW in low‐income countries (Clermont et al., 2018; Harding et al., 2017; Isanaka et al., 2019; Jones et al., 2021; Klevor et al., 2016; Young et al., 2015).

The objective of this present study was to identify the two most preferable BEP supplements among a larger group of 11 supplements specifically produced for use in pregnancy and lactation in the South Asian context for this study. In this study, we discuss the mixed‐methods design used to shortlist the most accepted BEP supplements among pregnant women and triangulate the quantitative and qualitative findings to discuss the facilitating factors and barriers to appropriate utilisation of the supplements.

## 2 METHODS

### 2.1 BEP supplements

To meet the nutritional needs of pregnant women in rural Nepal, 11 BEP supplements were developed in different flavours and forms specifically for this study according to the BMGF guidelines (Bill & Melinda Gates Foundation, 2017). Nine of the BEP supplements were developed in collaboration with Nutriset S.A.S. in France and two were developed in collaboration with MARS Incorporated in India. Out of the 11 BEP supplements, six were characterised as sweet and five as savoury. The ‘sweet’ products were not excessively sweet but sweeter in comparison to the savoury products. The added sugar in the sweet products ranged from 11.4 to 21.6 g per 100 g serving for the sweet products and 0.0 to 5.1 g for the savoury products (Table S1). Each BEP supplement's nutrient content was either already in line with the guidance for the macro‐ and micronutrient content developed by the experts' consultation meeting organised by the BMGF or was close and could be brought fully in line if selected (Bill & Melinda Gates Foundation, 2017). The nutrient composition of each of the 11 BEP supplements is presented in Table 1.

**Table 1:** Nutrient composition of the 11 BEP supplements per serving size

Nutrient	Sweet LNS	Sweet mango bar	Vanilla filled sticks	Sweet vanilla biscuit	Cocoa drink	Vanilla drink	Savoury LNS	Savoury masala bar	Savoury curry biscuit	Seasoned pillows	Unseasoned pillows
Energy (kcal)	549	303	476	318	312	318	547	309	415	321	303
Protein (g)	15	16	17	15	16	15	15	16	15.2	12	13
Lipids (g)	36.6	18	23.5	16	14	3.8	36.5	21	26.8	12	9
Calcium (mg)	581	560	523	560	560	697	592	560	512	31.5	31.5
Zinc (mg)	15	15	15	15	15	15	15	15	15	8	8
Copper (mg)	1.3	1.3	1.3	1.3	1.3	1.1	1.2	1.3	1.3	–	–
Iron (mg)	27	27	28	27	27	27	27	27	28	10	10
Iodine (μg)	248	250	216	250	250	242	249	250	211	–	–
Folic acid (μg)	397	400	398	400	400	402	400	400	392	90.4	90.4
Selenium (μg)	65	65	66	65	65	65	65	65	62	–	–
Manganese (mg)	2.2	2.1	2.2	2.1	2.1	1.7	2.1	2.1	2.6	–	–
Linoleic acid (g)	5.4	3.1	3.7	0.7	0.6	0.1	12.4	3.8	7.5	–	–
α‐Linolenic acid (g)	1.3	1.3	1.3	1.3	1.3	–	1.3	1.2	1.3	–	–
Vitamin A (μg)	764	770	767	770	770	774	770	770	746	400	400
Vitamin D (μg)	15	15	14	15	15	15	15	15	14	0.0065	0.0065
Vitamin E (mg)	16	16	16	16	16	16	16	16	16	–	–
Vitamin C (mg)	99	100	99	100	100	100	100	100	97	80	80
Vitamin B1 (mg)	1.4	1.4	1.4	1.4	1.4	1.4	1.4	1.4	1.4	0.8	0.8
Vitamin B2 (mg)	1.5	1.4	1.5	1.4	1.4	1.5	1.5	1.4	1.5	1.0	1.0
Vitamin B5 (mg)	6.9	7	7	7	7	7	7	7	6.9	–	–
Vitamin B6 (mg)	1.9	1.9	1.9	1.9	1.9	1.9	1.9	1.9	1.9	1.0	1.0
Vitamin B12 (μg)	2.6	2.6	2.6	2.6	2.6	2.6	2.6	2.6	2.5	2.0	2.0
Vitamin K (μg)	70	72	70	72	72	72	72	72	70	–	–
Niacin (mg)	18	18	18	18	18	18	18	18	18	10	10
Manufacturer	Nutriset	Nutriset	Nutriset	Nutriset	Nutriset	Nutriset	Nutriset	Nutriset	Nutriset	Mars	Mars

### 2.2 Study overview

The current study was the first of two phases of formative research designed to inform which BEP supplements among the 11 forms, types and flavours were the most acceptable in the South Asian context. This first phase was a single‐meal test that aimed to collect data to describe the general preferences/acceptability across different BEP supplement types and flavours and salient facilitating or constraining factors related to the use of each. Results from the first phase would identify a small number of BEP supplements for an 8‐week home‐tasting pilot trial (second phase). The shortlisted BEP supplements found to be most acceptable and utilised from this formative research would then be used in a larger randomised controlled trial (RCT) designed to test the efficacy of the selected BEP supplement(s) during pregnancy and lactation on pregnancy and child health outcomes in the same population (ClinicalTrials.gov Identifier: NCT03668977).

### 2.3 Study setting

This study was conducted in Sarlahi, a rural district located in the southern plains of Nepal. The study population in Sarlahi district meets the definition of an undernourished population based on the nutritional status of >28,000 pregnancies enroled in a previous large trial conducted in 2012–2017 (ClinicalTrials.gov, NCT01177111). Early pregnancy mean BMI among the study participants was 19.1 kg/m^2^ and 37% were underweight, with a BMI < 18.5 BMI (data not published). The incidence of LBW and SGA was 29.4% and 46.8%, respectively, in the same trial (data not published).

The typical daily diet in Sarlahi like in most parts of Nepal consists of rice (main staple), lentils and some seasonal vegetable dish with a pickle dish consumed twice daily by most people. Previous research in this study population showed that rice intake can be reduced during pregnancy, which could be attributed to an aversion to food or lack of appetite while for most protein and micronutrient‐rich foods, the majority reported eating the same quantities during pregnancy (Christian et al., 2006). Another study found that multiple micronutrient deficiencies are common during early pregnancy in this population (Jiang et al., 2005).

Recruitment for this first phase of the formative research was conducted in Pidari and Pipariya Village Development Committee (VDC; previous lowest administrative structure which was dissolved in March 2017 and replaced by rural municipalities) that fall under Haripur and Kabilasi municipalities. (‘Municipality’ in this context is an administrative term, but actually is comprises of a number of different VDCs). The study area within each municipality was selected because it was of moderate size (i.e., not too big or small a municipality in terms of geographical size), centrally located (i.e. middle of the district) and representative of the study district in terms of ethnicity, caste and religion. The two VDCs that were selected in those municipalities have both Hindus and Muslims representative of the study district which is a majority Hindu population and about 8% Muslim. The population in these areas, as opposed to the northern areas of the district, is more rural and consists mostly of the *Madhesi* (people from the Terai plains) ethnicity, which makes up the majority of Terai as opposed to the *Pahadis* (people from the hills). The ethnicity, caste and religion of our formative research population are generalisable to the Terai region as the sample group is similar to that in the Terai which is predominantly *Madeshi* ethnic group of Hindu religion who live in rural areas. We sampled more Muslims in our study than the current distribution in the population so as to get more in‐depth data on this minority group in the qualitative interviews since they would be included in the main trial.

The implementation of the data collection and field work was carried out by the Nepal Nutrition Intervention Project – Sarlahi (NNIPS), which is a long‐running research effort headed by investigators in the Department of International Health at Johns Hopkins Bloomberg School of Public Health in collaboration with Nepal Netra Jyoti Sangh, a national Nepali non‐governmental organization under the auspices of the Social Welfare Council of Nepal.

### 2.4 Recruitment and inclusion criteria

The inclusion criteria for enrolment of women into this study were to be married, pregnant and between 15 and 40 years of age. As per the Nepal Health Research Council research ethical requirement, a married woman under the age of 18 years is considered a minor and so was recruited and enroled in this study only after obtaining signed informed consent from the husband or guardian. Women were excluded if they had a known allergy to soy, dairy products, eggs, gluten or nuts. All women in the study area were recruited from the community; those who met the inclusion criteria and did not fit the exclusion criteria were added to the recruitment list which comprised 82 pregnant women. A purposive sample of 40 pregnant women was selected based on their age and gestational age and enroled into the study after obtaining informed consent. The first author (TPL) helped purposively sample the participants with consultation with the NNIPS senior field team staffs.

### 2.5 Study design and procedures

This formative research used a mixed‐methods approach combining qualitative and quantitative methods for data collection. Each participant participated in two consecutive days of data collection: the first day included individual tasting of six “sweet” supplements, and the second day included the tasting of five savoury BEP supplements followed by FGDs. On the first day, the interviewer went to the woman's home and collected basic socio‐demographic information from the participant. After that, the participant was asked to consume test portions of each of the six sweet BEP supplements. These were offered in a randomised sequence to avoid possible bias due to order of presentation. The test portions for each supplement were pre‐portioned by the field staff to be 25% of the weight of a full daily portion. The women were given up to 30 min to consume as much of each of the test portions as they could/wanted to or until they said they would not eat any more. After the consumption of the test portions of each BEP supplement, a structured questionnaire was used to ask the participant to rate the characteristics of each BEP supplement based on her perceptions of its organoleptic properties (i.e., colour, odour, taste, texture and overall liking) using a 7‐point Likert scale (1 = Dislike it very much to 7 = Like it very much). In addition, data were collected on willingness to consume these supplements every day during pregnancy and 6 months postpartum for up to a total of 12 months using a 5‐point Likert scale (1=Definitely would not eat every day to 5=Definitely would eat every day). Information as to whether the full portion size of each supplement was hypothetically the right amount, too much or too little for a daily snack portion was also collected. Data regarding the extent to which the product was ‘easy to prepare and consume’ and ‘easy to consume in between meals as a snack’ were collected separately using a 7‐point Likert scale (1 = Very difficult to 7 = Very easy). Women were also asked if they perceived the supplement as a food, a medicine or as both a food and medicine by directly asking her opinion given the three options. This question was in Likert‐scale format but was found to be very confusing for the participants during the pre‐test phase and so it was decided to keep it simpler. At the end of the first day, each participant was asked to rank the six sweet products from 6 (Most preferable/acceptable) to 1 (Least preferable/acceptable) according to each of the organoleptic properties. On the second day, each participant was brought to the NNIPS field office for tasting the test portions of the remaining five savoury products in a randomised sequence. The same structured questionnaire was administered on a one‐to‐one basis to assess each supplement's acceptability and the participant's willingness to use it in the future, followed by a ranking of the five savoury products on their organoleptic properties. An overall ranking of the top three most preferred out of the 11 BEP supplements was finally collected from each participant.

**Figure 1:**
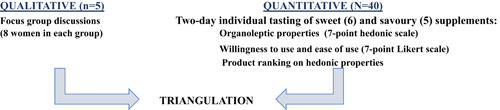
Mixed‐method study design overview

Complementary qualitative data were also collected using FGDs. Each of the 40 women was assigned to one of five FGD groups (A–E); the participants were divided into groups to promote homogeneity within each group in terms of age group (≤20 years and >21 years of age) and caste/religion (Muslims/low‐caste Hindu/high‐caste Hindu). In rural Nepal as in other South Asian countries, hierarchy in caste (upper and lower caste) among Hindus and religious affiliations (Hindu vs. non‐Hindu) needed to be considered so as to not affect the dynamics of interactions and discussions in an FGD setting (personal communication with senior staff at NNIPS who have worked for more than 30 years in health research, 2 July 2018). Similarly, age can also affect the dynamics within a group (Kumar, 1987); younger women may hesitate to speak freely in a group with older individuals given the South Asian norms of showing respect to elders by not speaking against them. The FGDs aimed to better understand factors influencing overall acceptability and preferences of flavour profiles, as well as sharing dynamics, local food practices and potential utilization of each of the 11 supplements. Focus groups generated data to better understand social norms. An additional ranking exercise was included in the FGDs to elicit further narratives around characteristics affecting the potential use of the products and how those characteristics related to each other. Participants were then asked to discuss and reach consensus as a group on their top three supplements. Figure 1 illustrates the mixed‐methods approach used to triangulate the data of this study.

Data collection was conducted over three weeks from 16 July to 1 August 2018. All data collection activities were conducted in the local language, Maithili. We trained four male interviewers to conduct the first‐day test meal tasting at the participant's homes mainly as the male interviewers rode motorbikes and could easily commute to the households. We also trained eight female interviewers to conduct the second‐day test meal tasting which took place at the office. This allowed all eight interviews to be conducted at the same time at the office. We further trained five of the eight female interviewers (who had previous experience with qualitative interviews) on the FGD guide and each one of them moderated an FGD.

During the training process, the quantitative and qualitative guides were pre‐tested with women in the study area and changes in terms of rephrasing certain questions were made. Training included going through the study procedure, questionnaire/guides and extensive practice through role play and field testing. Data for the quantitative forms were recorded using the Research Electronic Data Capture (REDCap, © Vanderbilt University) application on a password‐encrypted mobile android device. The FGDs were audio recorded, transcribed verbatim into Nepali using the notes and audio recordings by the moderator, and then translated into English for analysis.

### 2.6 Data analysis

Participant characteristics, including socio‐demographic and pregnancy characteristics, were summarised using means (*SD*) for continuous measures and using counts and proportions for discrete measures. The 7‐point hedonic scale responses rating sensory properties, and responses to the Likert‐scale responses for the themes ‘acceptability’, ‘perception of product use’ and ‘willingness to use for 12 months’ were presented as medians (interquartile range). Stata version 15.0 (StataCorp) was used for this analysis.

The results of the ranking exercise were summarised by each type of product grouping (sweet and savoury) and overall ‘Top 3’. In each ranking exercise, a supplement was assigned the highest score for the most preferred product and lowest score for the least preferred product. For the overall likeability ranking of ‘top 3’ BEP supplements out of the 11 supplements, a product was awarded three points if it was ranked first, two points if ranked second, one point if it was ranked third and zero points if it was not included in the top 3. The sum of each score for the 11 BEP supplements was then ranked using the sum of ranks method in Excel.

Preliminary iterative analysis of the qualitative data was conducted throughout the data collection process. In conclusion of the data collection, full analysis of the qualitative data was conducted using thematic analysis (Braun & Clarke, 2006). Dominant themes were identified through the systematic review of FGDs and field notes and a thematic framework was iteratively developed. Salient concepts were coded, and their occurrence and recurrence were labelled. Basic themes were deductively generated. These initial themes were reviewed and emergent codes and sub‐codes were iteratively refined throughout the process. The transcripts of the five FGDs were coded independently by two qualitative methods researchers (Katie Moore and Leslie Jones) using Dedoose software (Version 8.2.32; SocioCultural Research Consultants, 2016) and/or by hand. The decision to code by hand or using software was a matter of individual preference and was not believed to affect the substance of coding in any way. The same codebook was used by both coders and a small sample of transcripts was coded by each of two researchers to assess inter‐coder reliability. This approach was chosen due to time limitations and the simplicity of the coding structure, which did not require significant interpretation nor did it result in coding variation in the sample checked. The emerging trends were critically analysed according to the research objectives to assess which product types and varieties were preferred and why, what factors affected women's choice of preferred products, how those products would be incorporated into the usual diet, the acceptability of snacking and sharing, and the acceptability of at‐home consumption of products.

### 2.7 Ethical considerations

Written informed consent was obtained from all the respondents. In addition, signed informed consent from a guardian or husband was obtained for participants under 18 years of age as per the research ethics requirement of the local ethics review body. Ethical approval was obtained from The Johns Hopkins Bloomberg School of Public Health Institutional Review Board (Baltimore, USA), George Washington University (Washington D.C., USA) and the Nepal Health Research Council, Ministry of Health and Population (Kathmandu, Nepal).

## 3 RESULTS

The mean (*SD*) age of study participants was 22.7 (3.7) years and the ages ranged from 16 to 30 years. The religion of the majority of the participants was Hindu (72.5%) and their ethnicity was *Madeshi* (i.e., southern plains; 97.5%). The socio‐demographic and pregnancy characteristics of the study participants are presented in Table 2.

**Table 2:** Socio‐demographic and pregnancy characteristics of study participants

Socio‐demographic characteristics	*N* = 40
Age in years, mean (*SD*)	22.7 (3.7)
*Marital status, n (%)*	
Married and with husband	40 (100)
*Education background, n (%)*	
None	24 (60.0)
Primary (1–5)	4 (10.0)
Secondary (6–10)	10 (25.0)
Higher secondary	2 (5.0)
*Mean number of years (SD)*	*3.1 (4.3)*
*Religion, n (%)*	
Hindu	29 (72.5)
Muslim	11 (27.5)
*Ethnicity, n (%)*	
*Madeshi* (southern plains)	39 (97.5)
*Pahadi* (hill)	1 (2.5)
*Pregnancy characteristics*	
First pregnancy, *n* (%)	11 (27.5)
Number of living children, mean (*SD*)	2.1 (1.2)
Gestational age in months, mean (*SD*)	5.4 (1.9)
Number of antenatal consultations, mean (*SD*)	2.2 (1.8)

### 3.1 Hedonic properties

Table 3 shows the results of the hedonic scale responses rating the sensory properties (colour, taste, smell, texture and overall) of the 11 BEP supplements. The sweet LNS, sweet vanilla drink, savoury LNS and savoury seasoned pillows were rated highly, with a median response of ‘7 = Like very much’ on all sensory properties. The taste, smell and/or texture of the sweet mango bar, sweet cocoa drink, savoury masala bar and savoury curry biscuit received lower ratings; the lower quartile (Q1) of the interquartile range for the latter BEP supplements show several scores between 1 (dislike very much) and 3 (dislike slightly; Table 3). The distribution of the responses on the 7‐point hedonic scale showed that sweet LNS and savoury seasoned pillows were liked very much by the vast majority of the participants (75% and 70%, respectively). In contrast, 30% and 35% of the participants reported disliking the sweet cocoa drink and savoury curry biscuits very much, respectively (Table 3). Further analysis to assess for variation in responses to the sensory properties by participants socio‐demography characteristics: age (<21 years, 21–25 years and 26–30 years), gestational age (three trimesters), education (no schooling and any schooling) and religion (Muslim and Hindu) found no significant variation (data not shown). This may be attributed to the small sample size.

**Table 3:** Hedonic scales results for all 11 BEP supplements

	Sweet LNS		Sweet mango bar	Vanilla filled sticks	Vanilla biscuit	Cocoa drink	Vanilla drink	Savoury LNS	Savoury masala bar	Savoury curry biscuit	Seasoned pillows	Unseasoned pillows
**Hedonic scale scores, median (IQR) scores based on 7‐point Likert scale (1** = **Dislike very much to 7** = **Like very much)**												
Colour	7 (6, 7)	6 (5, 7)		7 (6, 7)	7 (6, 7)	6 (5, 7)	7 (6, 7)	7 (6, 7)	7 (5.5, 7)	6 (2, 7)	7 (6.5, 7)	6 (5.5, 7)
Taste	7 (6, 7)	5.5 (1.5, 7)		7 (6, 7)	7 (6, 7)	5 (1, 7)	7 (5, 7)	7 (6, 7)	6 (3, 7)	5 (1, 7)	7 (6, 7)	6 (3, 7)
Smell	7 (6, 7)	6 (2, 7)		6 (5.5, 7)	7 (6, 7)	6 (4, 7)	7 (6, 7)	7 (6, 7)	6 (3, 7)	5.5 (1,7)	7 (6, 7)	6 (5, 7)
Texture	7 (6, 7)	6 (1.5, 7)		7 (6, 7)	6 (5.5, 7)	5 (1, 7)	7 (5.5, 7)	7 (6, 7)	6 (4, 7)	5 (1, 7)	7 (6, 7)	6 (5, 7)
Overall appreciation	7 (6.5, 7)	5 (2, 7)		6 (5, 7)	7 (6, 7)	5 (1, 7)	7 (5.5, 7)	7 (6, 7)	6 (5, 7)	6 (1, 7)	7 (6, 7)	6 (5, 7)
Perceived child likeability	7 (6.5, 7)	6 (4, 7)		7 (7, 7)	7 (7, 7)	6 (3, 7)	7 (6, 7)	7 (6, 7)	6 (5, 7)	5 (3, 7)	7 (7, 7)	7 (5, 7)
Perceived adult likeability	7 (6, 7)	6 (4, 7)		6 (6, 7)	7 (5, 7)	4.5 (3.5, 6)	7 (5, 7)	7 (6, 7)	6.5 (5, 7)	5 (2.5, 7)	7 (6, 7)	6 (5, 7)
**Distribution of overall likeability responses on 7‐point Likert scale**												
Liked very much	75.0%		35.0%	45.0%	52.5%	32.5%	62.5%	57.5%	47.5%	30.0%	70.0%	35.0%
Liked moderately	7.5%		12.5%	25.0%	25.0%	12.5%	12.5%	22.5%	12.5%	22.5%	15.0%	22.5%
Liked slightly	7.5%		22.5%	20.0%	12.5%	17.5%	10.0%	12.5%	17.5%	10.0%	10.0%	22.5%
Neither like/dislike	5.0%		0%	0%	0%	0%	0%	2.5%	0%	0%	0%	2.5%
Dislike slightly	0%		2.5%	2.5%	2.5%	2.5%	2.5%	0%	0%	2.5%	0%	2.5%
Dislike moderately	0%		7.5%	0%	2.5%	5.0%	2.5%	0%	2.5%	0%	0%	2.5%
Dislike very much	5.0%		20.0%	7.5%	5.0%	30.0%	10.0%	5.0%	20.0%	35%	5.0%	12.5%

The qualitative findings from the FGDs were consistent with the quantitative sensory assessment. Participants commented favourably on the sweet LNS having a sweet/salty flavour mix, and contrasted it to the savoury LNS which, though well‐liked, was not as popular as the sweet version due to what some perceived as excessive salt. Participants likened the sweet LNS to a number of favourite local foods, including ‘*Halwa’* a sweet pudding, ‘*Satu*’, a cereal‐based snack, and a variety of commercial products. Women consistently liked the savoury seasoned pillows for colour and for the taste, which many described as ‘a little salty and a little sweet’ but also ‘a little spicy’. One response from a woman in FGD group A was illustrative: ‘*Yes, its sour and spicy type, that's why they would eat more during pregnancy’*. The seasoned pillows were positively associated with a number of other familiar tasting foods, particularly Indian crisps and snacks. The vanilla drink also had positive reviews during the FGDs, where most of the participants liked its colour, smell and taste. One person in FGD group B commented that the smell resembled that of ice cream, while the taste was positively likened to peanut ‘*satu*’ and baby food or malted milk powders. In FGD group C, the vanilla drink was also likened to a powdered protein drink for pregnancy and lactation available in the local market. The vanilla biscuit was liked by most of the women in the FGDs, although some participants in FGD group C did not like its taste and smell and suggested it tasted like medicine.

The FGDs also highlighted the reasons for not liking certain properties of the BEP supplements. For instance, some participants in FGD group B disliked the savoury masala bar for its taste and smell. The texture was also described negatively as something that felt ‘rough’ and ‘sticks to your teeth’. The savoury curry biscuit was also disliked for its colour, taste and texture where participants in group B made specific and negative reference to individual flavours in the product, such as cumin, pepper, fenugreek, turmeric and *‘jwano’* (thyme seed), and commented that it was too salty and tasted like medicine as it was bitter to taste. A few women liked the cocoa drink but most did not, because of its colour and bitter taste, which was compared to medicine. Some of the most negative comments on the cocoa drink came from participants in FGD group B: *‘It tastes bitter and it's black’*; ‘*Just looking at the colour might make people feel like vomiting. It looks like sewage water*’. Participants disliked the unseasoned pillows because of its bland taste, although they found it similar to popcorn in terms of taste and smell.

### 3.2 Use of products during pregnancy

We explored perceptions of supplement use during pregnancy by asking, for each BEP supplement, how easy it would be to prepare and use and how easy it would be to eat as a snack in between meals. The responses to both questions were positive with the median response being ‘6 = Easy’ or ‘7 = Very easy’ for all 11 supplements (Table 4). There was little variability in the response to the question on “willingness to use the supplement daily for up to 12 months if provided for free’, with the median response for all 11 BEP supplements being ‘5 = Definitely would eat every day’. The median response to perceived likelihood of sharing the supplements with others was ‘1 = Definitely would not share’ (Table 4). Except for the cocoa and vanilla drink supplements, the majority of participants considered the supplements to be a food rather than a medicine as shown in Table 4.

**Table 4:** Women's perception of product use for all 11 BEP supplements

	Sweet LNS	Sweet mango bar	Vanilla filled sticks	Sweet vanilla biscuit	Cocoa drink	Vanilla drink	Savoury LNS	Savoury masala bar	Savoury curry biscuit	Seasoned pillows	Unseasoned pillows
**Perception of product use, median (IQR) scores (1** = **Very difficult to 7** = **Very easy)**											
Supplement is convenient to eat easy to prepare and use	7 (7, 7)	6 (5, 7)	7 (6, 7)	7 (6, 7)	6 (5.5, 7)	7 (6, 7)	7 (6, 7)	7 (6, 7)	7 (5, 7)	7 (7, 7)	7 (6, 7)
Supplement is easy to eat between meals as a snack	7 (6, 7)	6 (5, 7)	6 (6, 7)	7 (6, 7)	6 (5, 7)	7 (6, 7)	7 (6, 7)	7 (6, 7)	6.5 (5, 7)	7 (6, 7)	7 (6, 7)
**Willingness to use daily for 12 months if provided for free, median (IQR) scores (1** = **Definitely would not eat every day to 5** = **Definitely would eat every day)**											
Would use daily if provided for free	5 (5, 5)	5 (3, 5)	5 (5, 5)	5 (5, 5)	5 (3.5, 5)	5 (5, 5)	5 (4, 5)	5 (4, 5)	4.5 (4, 5)	5 (4, 5)	5 (4, 5)
**Likelihood of sharing product with others, median (IQR) scores (1** = **Definitely would not share to 5** = **Definitely would share)**											
Would share with others	1 (1, 4)	1 (1, 4)	1 (1, 2.5)	1 (1, 4)	1 (1, 4)	1 (1, 2)	1 (1, 4)	1 (1, 3.5)	1 (1, 4)	1 (1, 2.5)	1 (1, 4)
**Consider product to be a medicine or food or both or neither, *n* (%)**											
Medicine	9 (22.5%)	14 (35.0%)	8 (20.0%)	8 (20.0%)	18 (45.0%)	14 (35.0%)	7 (17.5%)	6 (15.0%)	9 (22.5%)	7 (17.5%)	10 (25.0%)
Food	17 (42.5%)	17 (42.5%)	17 (42.5%)	18 (45.0%)	9 (22.5%)	13 (32.5%)	18 (45.0%)	18 (45.0%)	17 (42.5%)	21 (52.5%)	18 (45.0%)
Both a medicine and food	14 (35.0%)	9 (22.5%)	15 (37.5%)	14 (35.0%)	13 (32.5%)	13 (32.5%)	15 (37.5%)	16 (40.0%)	13 (32.5%)	11 (27.5%)	12 (30.0%)
Neither a medicine nor food	0 (0%)	0 (0%)	0 (0%)	0 (0%)	0 (0%)	0 (0%)	0 (0%)	0 (0%)	1 (2.5%)	1 (2.5%)	0 (0%)
**Acceptability of portion size (for a snack), *n* (%)**											
Right amount for a daily snack	36 (90.0%)	33 (82.5%)	34 (85.0%)	37 (92.5%)	31 (77.5%)	28 (70.0%)	35 (87.5%)	33 (82.5%)	33 (82.5%)	33 (82.5%)	35 (87.5%)
Less than I would want as a daily snack	0 (0%)	2 (5.0%)	1 (2.5%)	2 (5.0%)	0 (0%)	1 (2.5%)	0 (0%)	0 (0%)	5 (12.5%)	1 (2.5%)	2 (5.0%)
More than I would want as a daily snack	4 (10.0%)	5 (12.5%)	5 (12.5%)	1 (2.5%)	9 (22.5%)	11 (27.5%)	5 (12.5%)	7 (17.5%)	2 (5.0%)	6 (15.0%)	3 (7.5%)

For each supplement type, the FGD facilitator probed the reasons why women would or would not use the supplements during the pregnancy period. Most of the participants agreed that they would eat the supplements every day during pregnancy as it would benefit the mother and baby, and they associated it with giving energy to the mother and baby. A participant in FGD group D stated, ‘*If you eat this, nothing will happen to the mother and child, they won't be weak, it will be good*’. In FGD group C, women expressed the intention to eat the product every day but stated that ‘*Sometimes it can be missed but will eat…*’ and that ‘*It will be missed during fasting period and all otherwise it can be eaten regularly*’. A few women had a strong aversion to the smell or taste of certain supplements like the mango bar, cocoa drink, vanilla biscuit, curry biscuit, and masala bar; they said the product(s) made them feel like vomiting and they would not be able to eat them every day.

Cost was raised as a potential obstacle to eating a supplement every day: ‘*If it is provided for free you can eat it [for 12 months] but cannot afford to buy it*’ (participant in FGD group A). When asked about convenience of eating the supplements, women did not identify any difficulties except for potential issues eating when others are around. One woman in group A said ‘*How to eat in front of many people, it does not look good, one person is eating and rest of others are looking*’. It was also suggested that it could be difficult to eat the supplements in front of children, who might expect them to share it.

In two of the FGD groups (A and B), the participants raised concerns about being limited to the use of one product for the duration of pregnancy. The majority of participants in both groups agreed that they could eat supplements throughout pregnancy, although it was highlighted that alternating between more than one supplement would be preferable. One woman in group B said ‘*eating the same food every day one will feel like vomiting* (‘*wak wak’*)…*look, people just don't eat rice and lentils every day, they sometimes eat vegetables and rice, don't they? It is possible to eat for one week [then] alternate’*. It was suggested by these women that ‘desires’ would not be fulfilled if the same products were eaten daily, and there was general agreement that products should be alternated weekly.

### 3.3 Ranking results

Table 5 presents the results of the (1) individual product ranking activity based on overall preference, (2) group product ranking activity and (3) the mean individual product acceptability score for the top five products along any of those three metrics. According to the individual ‘top 3’ ranking on overall preference and the mean score for product acceptability, the top two products were the sweet LNS and the seasoned pillows. The FGD group ranking indicated the sweet LNS as the most preferred, followed by the vanilla drink, and a tie between the seasoned pillows and vanilla biscuit for the third most preferred. The three least preferred BEP supplements based on the individual ranking of the ‘top 3’ were the unseasoned pillows, curry biscuit and cocoa drink with a sum of ranks score of 1, 6 and 6, respectively.

**Table 5:** Top five products across three primary metrics

	Sweet LNS	Seasoned pillows	Vanilla drink	Savoury LNS	Vanilla biscuit
Individual product ranking (points)	1 (51)	2 (43)	3 (37)	4 (34)	5 (28)
Group ranking (points)	1 (14)	3 (4)	2 (8)	5 (0)	3 (4)
Product acceptability (mean score on 7‐point scale)	2 (6.3)	1 (6.4)	5 (5.9)	3 (6.2)	4 (6.0)

## 4 DISCUSSION

This study assessed acceptability and preferences for 11 fortified BEP supplements among pregnant women and explored ideas about their consumption during pregnancy. Our quantitative and qualitative results suggest that the sweet LNS and savoury seasoned pillows had the highest acceptability in our study population.

Participants responded more negatively to some of the BEP supplements (the sweet cocoa drink, sweet mango bar, savoury curry biscuit and the savoury masala bar) in terms of overall likeability, taste and smell. Previous studies have shown that pregnant women's responses to supplements' sensory characteristics are key predictors of acceptability and adherence. The Rang‐din Nutrition study conducted in Bangladesh, which assessed acceptability and adherence among PLW to a 20‐g Lipid‐based Nutrient Supplement similar to the sweet LNS assessed in this study, showed that ‘bad smell of supplement’ and ‘feeling nausea or vomiting’ were responses most common among those with low adherence (Harding et al., 2014). Another acceptability study of a peanut‐based ready‐to‐use therapeutic food given to malnourished PLW in Bangladesh reported that, despite a perceived therapeutic benefit, almost 80% of the participants found it to be unacceptable due to its undesirable taste and smell (Ali et al., 2015). Sensory/organoleptic attributes of the supplements were central to acceptability of similar small‐quantity LNS given to PLW in two randomised trials in Ghana and Malawi (Klevor et al., 2016) and an acceptability study in rural Niger (Isanaka et al., 2019). In a trial among pregnant women in Mexico, micronutrient powders and tablets were preferred over fortified food (Nutrivida) because the participants disliked certain sensory properties of the fortified food (i.e., smell, taste and texture; Young et al., 2010).

The efficacy of a complementary feeding intervention depends on sustained consumption of the products (i.e., adherence). During pregnancy, women are more likely to have strong preferences for and aversions to certain smells and tastes (sweet vs. savoury), in addition to nausea and vomiting common in many pregnancies (Bowen, 1992). This study found that some pregnant women preferred sweet products and some savoury, so having at least one supplement of each kind would be likely to increase adherence among a group of pregnant women. The qualitative findings also highlighted the need for an option to choose different types of supplements on a weekly basis, especially when having to take the supplement regularly for a long period of time (i.e., daily use for 12 months starting from second trimester of pregnancy through the first 6 months of lactation). Very few efficacy trials of BEP supplements have provided an option to choose between two or more supplements; this could be an important strength in future intervention trials to ensure adequate long‐term adherence.

Similar to other acceptability studies of food supplements for PLW, one of the motivating factors for acceptance and possible adherence to the BEP supplements in this study was their perceived positive health benefit for the mother and the unborn baby (Adu‐Afarwuah et al., 2011; Clermont et al., 2018; Harding et al., 2014; Klevor et al., 2016; Mridha et al., 2012). Responses to questions about the likelihood to share or expectations to share the supplements with others in the household in a hypothetical situation were mixed; some women said they would have to share, especially with children, and others reported they would not. Sharing behaviour was further explored in the home‐tasting pilot trial (phase 2) that showed some sharing in both supplement type groups with an average sharing of no more than one day in the 8‐week testing period (Lama et al., 2021). In the pilot trial, we tried to ensure low sharing of supplements by advising the pregnant women that the supplements were for pregnant women only and also labelled the supplements with a picture of pregnant and lactating women. The majority of women who shared, reported sharing the supplement with their child or children in the household in the first week of the pilot trial (Lama et al., 2021). In‐depth interviews with the women and family members, indicated that referring to the supplements as medicine for pregnant women or eating the supplement when alone helped dissuade children from asking to share (Lama et al., 2021). Results of other home‐tasting pilot trials of maternal nutritional supplements support our findings that some sharing is expected. This has been seen especially in the beginning of trials, to respond to the curiosity of family or friends (Klevor et al., 2016) and particularly small children (Clermont et al., 2018). Sharing of food supplements among household members has been identified as a potential barrier to optimal supplement adherence (Beckett et al., 2016; Kodish et al., 2017). The majority of our study population identified the BEP supplements as a ‘food’ or ‘both food and medicine’, which is consistent with other food supplementation studies (Adu‐Afarwuah et al., 2011; Clermont et al., 2018; Mridha et al., 2012). A trial in Niger of three nutritional supplements found that women in the LNS arm were more likely to over‐consume the product because they associated it with food, compared to women in the other two arms who were given either a multi micronutrient supplement or iron/folic‐acid supplement (Clermont et al., 2018). This should be further explored in the larger efficacy trial. Similar formative research to understand PLW acceptability of BEP supplements was also implemented in Burkina Faso before a similar home‐tasting pilot trial also designed to inform a randomised trial to test the efficacy of BEP supplements on newborn and infant health and growth outcomes (Jones et al., 2021). The formative research in Burkina Faso was also conducted among 40 pregnant women and tested 12 different products, out of which 8 were the same products used in our study. The four different products in the Burkina Faso study were (i) fermented drink, (ii) tomato and onion bar, (iii) tomato and onion biscuit and (iv) chicken soup. Several of the flavours were developed specifically to evoke familiar flavours from the Burkinabè diet, such as the fermented drink. In contrast to our study, the pregnant women in the Burkina Faso site preferred products with familiar flavours that are perceived as sweet rather than savoury. The sweet LNS and sweet vanilla biscuit were the top two products and none of the savoury products was ranked in the top five product preferences (Jones et al., 2021).

The hedonic test and ranking exercise held over two days allowed us to quantify the responses on the organoleptic properties, rank the top three supplements on those properties and understand acceptability from multiple angles and perspectives. The FGD done at the end of the test meals provided further insight into the reasons for the high or low acceptance of the various supplements. By using both quantitative and qualitative data collection methods on the same topic, our study design included methodological triangulation. However, we did not use different participant types, which resulted in some drawbacks. During the FGDs, the discussion of each of the 11 supplements on the same topics turned out to be long and repetitive for the interviewers and the participants. So, one modification that could be made is restricting the FGD questions to only the top three or five supplements from the ranking exercise in the individual hedonic testing when involving the same participants. Another approach would be to conduct additional in‐depth interviews among health workers from the community such as female community health volunteers and auxiliary nurse midwives who are government health workers who promote and provide antenatal and postnatal care services respectively. Similar to a study conducted in Malawi to understand the preference of nutritional supplements among people living with HIV (Rodas‐Moya et al., 2016), interviewing health care workers to gain their perspective on the target population's preference and acceptance of the supplements can help triangulate data sources and further validate the findings.

One of the notable strengths of this study is the use of mixed methods to understand the acceptability of 11 BEP supplement prototypes. The combination of the quantitative hedonic test questions with the qualitative FGDs with the same participants allowed for an in‐depth understanding and exploration of the reasons for liking and not liking each of the supplement types along with triangulation of the findings to come to a clearer conclusion. Unlike other acceptability studies that nested their assessments within large longitudinal efficacy trials that were testing only one to two nutritional food supplements (Clermont et al., 2018; Harding et al., 2014; Klevor et al., 2016; Young et al., 2010), our study was uniquely positioned to test 11 prototypes of BEP supplements that were developed for PLW before an efficacy trial. This gave us the opportunity to identify the most acceptable BEP supplement(s) for use in the home‐tasting pilot trial (phase 2) which would then aim to assess midterm adherence for the shortlisted BEP supplements up to 8 weeks.

This study has some limitations. First, the test‐meal observation was conducted only once and so was subject to reactive behaviours (Gittelsohn et al., 1997); this could have been reduced through repeated observation of the test meals on consecutive days as done in the study in Niger (Isanaka et al., 2019). Second, social desirability bias may have resulted in underreporting of negative responses to attributes of the BEP supplements (Fisher, 1993). The Likert scores on organoleptic properties varied only slightly between products, with predominantly positive median scores in the 6 (Liked moderately) to 7 (Liked very much) range. This may reflect the limitations in using a Likert scale to assess supplement acceptability in a cultural context where people are unfamiliar with the Likert scales and may be unwilling to provide negative feedback (Flaskerud, 2012; J. W. Lee et al., 2002). The use of mixed methods to triangulate the findings using hedonic scales, ranking exercises, and FGDs aimed to mitigate this bias to some extent. In addition, some products did appear to be disliked by at least some fraction of the participants relative to other products, allowing for differentiation of products in terms of preferences. Certain questions were more hypothetical questions (e.g., would you consume these supplements every day during pregnancy and six months postpartum?) making it difficult to answer and could have resulted in some bias. However, the second phase of the formative research with the 8‐week home‐tasting trial will assess longer‐term use and acceptability with the shortlisted BPE supplements. Another major limitation to this study was that since we grouped the FGDs by caste/religion and age resulting in five strata (Table S2), and each stratum consisted of one FGD only due to small sample size of the study (*N* = 40), the authors cannot confirm that data saturation of key themes among all strata was reached. Finally, the second‐day test‐meal observation and the FGDs were conducted at the office premises maintaining privacy and confidentiality but the change in interview location to an outside of home setting could potentially have influenced the results. However, this cannot be ascertained from the data.

## 5 CONCLUSION

This study assessed the acceptability of 11 BEP supplements among pregnant women in Nepal. The fortified BEP supplements were developed for this study specifically to meet the nutritional needs of pregnant and lactating women. Understanding the acceptability of such food supplements before their use among PLW is vital to ensure regular consumption. We conclude that the sweet LNS and savoury seasoned pillows were the most acceptable to pregnant women in this rural Nepali population.

## CONFLICT OF INTERESTS

The authors declare no conflict of interest.

## ETHICS STATEMENT

The Johns Hopkins Bloomberg School of Public Health Institutional Review Board (Baltimore, USA), George Washington University Institutional Review Board (Washington D.C., USA) and the Nepal Health Research Council, Ministry of Health and Population (Kathmandu, Nepal) reviewed and approved this study.

## AUTHOR CONTRIBUTIONS

Saskia de Pee, Sheila Isanaka, Juliet Bedford, Leslie Jones and Katie Moore conceived the study design and developed the study tools. All authors contributed towards the review and finalisation to the study design and study tools. Tsering P. Lama, Katie Moore, Leslie Jones and Subarna K. Khatry were involved with the training of the data collections. Tsering P. Lama and Subarna K. Khatry led the field team and supervision of the data collection. Tsering P. Lama analysed the quantitative data and prepared the first draft of the manuscript. Katie Moore and Leslie Jones analysed the qualitative data. Sheila Isanaka, James M. Tielsch, Joanne Katz, Saskia de Pee, Steven C. LeClerq and Juliet Bedford were involved in the interpretation of the data. All authors were involved in the critical revisions of the manuscript for important intellectual content. All authors approved the final draft of the manuscript.

### 1 DATA AVAILABILITY STATEMENT

The data are not shared in this manuscript but deidentified data can be made available upon request.

## Supporting information

Supporting information.

Supporting information.
