# Supplementary material for: Acceptability of 11 fortified balanced energy‐protein supplements for pregnant women in Nepal
Source: Matern Child Nutr. 2022 Mar 9;18(3):e13336. doi: 10.1111/mcn.13336 (PMC9218317; doi:10.1111/mcn.13336)
Supplement: Supplementary file 1 — Supporting information. [file MCN-18-e13336-s002.docx]

Supplementary Table 1

Table S1: Added Sugars per 100g serving

| **Product Name** | **Added Sugars/100g** |
| --- | --- |
| Sweet lipid-based spread | 18.0g |
| Sweet Mango bar | 21.6g |
| Vanilla Filled sticks | 20.0g |
| Sweet Vanilla biscuits | 19.9g |
| Vanilla drink | 11.4g |
| Cocoa drink | 19.3g |
| Tomato and onion lipid-based spread | 2.0g |
| Savory Masala bar | 0.0g |
| Savory Curry biscuits | 0.0g |
| Seasoned pillows | 5.1g |
| Unseasoned pillows† | 3.9g |
