# Supplementary material for: Acceptability of 11 fortified balanced energy‐protein supplements for pregnant women in Nepal
Source: Matern Child Nutr. 2022 Mar 9;18(3):e13336. doi: 10.1111/mcn.13336 (PMC9218317; doi:10.1111/mcn.13336)
Supplement: Supplementary file 2 — Supporting information. [file MCN-18-e13336-s001.docx]

Supplementary Table 2: The demographic composition of each FGD group

|  | **FGD Group A** | **FGD Group B** | **FGD Group C** | **FGD Group D** | **FGD Group E** |
| --- | --- | --- | --- | --- | --- |
| **Age** | >21 years | <=21 years | >21 years | <= 21 years | >20 years |
| **Caste/religion** | Muslim only | Hindu higher caste | Hindu higher caste | Mix of Hindu low caste and Muslim | Hindu lower caste |
